# Factors influencing recurrence after complete remission in children with hepatoblastoma: A 14-year retrospective study in China

**DOI:** 10.1371/journal.pone.0259503

**Published:** 2021-11-29

**Authors:** Fan Li, Weiling Zhang, Huimin Hu, Xia Zhu, Yi Zhang, Dongsheng Huang

**Affiliations:** Department of Pediatrics, Beijing Tongren Hospital, Capital Medical University, Beijing, China; Cincinnati Children’s Hospital Medical Center, UNITED STATES

## Abstract

**Objective:**

After a complete remission to treatment for hepatoblastoma, some children still have recurrence. We identified and explored the factors that influence recurrence after complete remission in a retrospective study.

**Methods:**

Of 197 children with hepatoblastoma, 140 (71.1%) achieved initial complete remission and were enrolled in factor analysis. Variables consisted of age, sex, PRE-Treatment EXTent of tumor (PRETEXT) stage, pathologic type, metastatic disease, serum alpha-fetoprotein level, vascular involvement, and surgical margin status. We employed univariate and multivariate analyses to assess the relationship between each factor and tumor recurrence.

**Results:**

Of 140 children who achieved initial complete remission, 42 (30%) had recurrent hepatoblastoma. The 5-year overall survival rates for the non-recurrence and recurrence group were 99.0% and 78.6%, respectively. The overall 1-year, 3-year, and 5-year recurrence-free survival (RFS) rates were 77.8%, 69.8%, and 69.8%, respectively. All recurrences occurred within 2 years from complete remission. The RFS rate was significantly higher in children younger than 3 years and in those with mixed pathological type, PRETEXT II and III, without metastatic disease, without vascular involvement, and microscopic negative margin than in that of children older than 3 years, with epithelial pathological type, PRETEXT IV, metastatic disease, vascular involvement, and macroscopic positive margin (*P* < 0.001, = 0.020, < 0.001, = 0.004, = 0.002, and < 0.001, respectively). The independent risk factors for recurrence after complete remission were age ≥3 years, PRETEXT IV, and metastatic disease (*P* < 0.05).

**Conclusion:**

Age, PRETEXT stage, metastatic disease, vascular involvement, pathologic type, and surgical margin status might be associated with recurrent hepatoblastoma after complete remission; meanwhile, age ≥3 years, PRETEXT IV, and metastatic disease are independent risk factors of recurrence. Further research is needed on the causes of tumor recurrence, which may improve the long-term outcomes of children with hepatoblastoma.

## Introduction

Hepatoblastoma is a rare pediatric solid tumor. Although it has been reported to account for only approximately 1% of all childhood malignancies, it is still the predominant primary hepatic malignancy in children [1]. With improved treatment regimens, the progressive improvement in prognoses over recent decades has outpaced that of most other solid tumors. The 5-year survival rate for children with metastatic hepatoblastoma rose steadily from 27% reported in the 1990s to 79% reported in 2013 [2,3].

Although a significant number of children achieve a complete or partial remission after a combination of surgery and chemotherapy, approximately 20% of children with hepatoblastoma still develop recurrence [4–6]. Once a recurrence occurs, the case fatality rate increases significantly. It has been reported that the 3-year event-free survival (EFS) rate and the overall survival (OS) rate in children with recurrent hepatoblastoma is only 34% and 43% [6], respectively.

In recent years, with the improved understanding of prognostic factors [7–10], treatment regimens and risk stratification methods have sought to improve the EFS and OS rates of children with hepatoblastoma [11,12]. To date, however, there has been little research on how to effectively prevent recurrence after a complete remission (CR). Although tumor recurrence had been hypothesized to be a poor prognostic factor, the factors themselves that lead to the recurrence of hepatoblastoma after CR are not yet well understood. Further study is needed to identify the factors associated with an increased risk of recurrence. By adjusting the initial treatment for children with high risk factors of recurrence, it is possible to reduce the risk of recurrence after CR and thus improve survival.

Therefore, we explored the possible factors affecting recurrence through a retrospective study of children with first CR after treatment, with the idea of improving long-term recurrence-free survival (RFS) in children with hepatoblastoma.

## Materials and methods

### Clinical materials

This study was carried out following the rules of the Declaration of Helsinki, and was approved by the Ethics Committee of Beijing Tongren Hospital, Capital Medical University, Beijing, China. Written informed consent for publication of information without disclosing any information related to the identity of the subject was obtained from all children’s guardians. There was no central review of the study.

This study was a retrospective observational cohort study that began in 2019, which included children with hepatoblastoma who were hospitalized in department of pediatrics between March 2005 and December 2018. The exclusion criteria were: (1) children without surgery or without surgical pathological results, (2) children with discontinued or abandoned treatment, (3) children who were not initially treated in our hospital, (4) children with incomplete medical records.

The variables included in the study were age, sex, PRE-Treatment EXTent of tumor (PRETEXT) stage, pathologic type, metastatic disease, serum alpha-fetoprotein (AFP) level (assessed at diagnosis), vascular involvement (hepatic/portal vein or vena cava), and surgical margin status. PRETEXT staging is based on Couinaud’s segmentation system of the liver [13]. In PRETEXT I, the tumor burden is limited to 1 section; in PRETEXT II, 1 or 2 sections are involved and 2 adjoining sections are tumor free; in PRETEXT III, 2 or 3 sections are involved, and no 2 adjoining sections are tumor free; in PRETEXT IV, all 4 sections are involved [14]. The PRETEXT staging results were determined by computed tomography (CT) imaging results. The ages were grouped according to the unified risk classification from the Children’s Hepatic Tumors International Collaboration (CHIC) consortium [11], which uses 3 age groups: younger than 3 years, 3 to 7 years, and 8 years and older. Given there were only 5 patients in the group older than 8 years, this group was combined with the 3–7-year-old group. According to CHIC, serum AFP levels were divided into groups of less than 100 ng/mL, 100–1000 ng/mL, and greater than 1000 ng/mL. Given there were only 2 cases with serum AFP less than 100 ng/mL, these were combined with the 100–1000 ng/mL group. The serum AFP levels of all children were determined by chemiluminescence immunoassay. Based on the international pediatric liver tumor consensus classification [15], cases were divided into epithelial (fetal, embryonal, macrotrabecular, small-cell, cholangioblastic, and mixed subtypes) and mixed (stromal derivatives and teratoid) types. Pathological results of all children were reported by the department of pathology of the cooperative hospital. Metastatic disease was defined as non-contiguous tumor spread found by imaging examination, including lung metastases, bone metastases, intracranial metastases, pleural metastases, and implantation metastases (intestinal or diaphragmatic metastases), which were comprehensively determined by multi-site CT, ultrasound and bone scanning. Pulmonary metastases were confirmed by at least two radiologists, which were not defined with reference to the AHEP-1531 and AHEP-0731 studies [16,17]. According to International Childhood Liver Tumor Strategy Group (SIOPEL), vascular involvement was annotated as ‘V’ (all three hepatic veins or the vena cava) or ‘P’ (portal bifurcation or the main portal vein), which were detected by CT, ultrasound, or during surgery. The status of surgical margin was obtained from the surgical records and histopathological reports, which were classified as MacroPM (Macroscopically positive margin), MicroPM (Microscopically positive margin), and MicroNM (Microscopically negative margin). A MacroPM was defined as the presence of macroscopic tumor invasion at the surgical margin, and a MicroPM was defined as the presence of microscopic tumor invasion at the surgical margin. CR was defined as the disappearance of all known disease, determined by 2 observations not less than 4 weeks apart, while tumor recurrence was defined as reappearance of disease after complete eradication [18]. A tumor recurrence was considered if a definite new lesion was found on imaging or if a suspected new lesion was found accompanied by at least 1-log increase in serum AFP level. All patients with recurrent lesions required a biopsy or secondary surgical resection to confirm the reappearance of disease. The time of recurrence was dated from the first positive record by imaging examination [18]. RFS was defined as the time from CR to tumor recurrence.

### Treatment strategy

**Fig 1:**
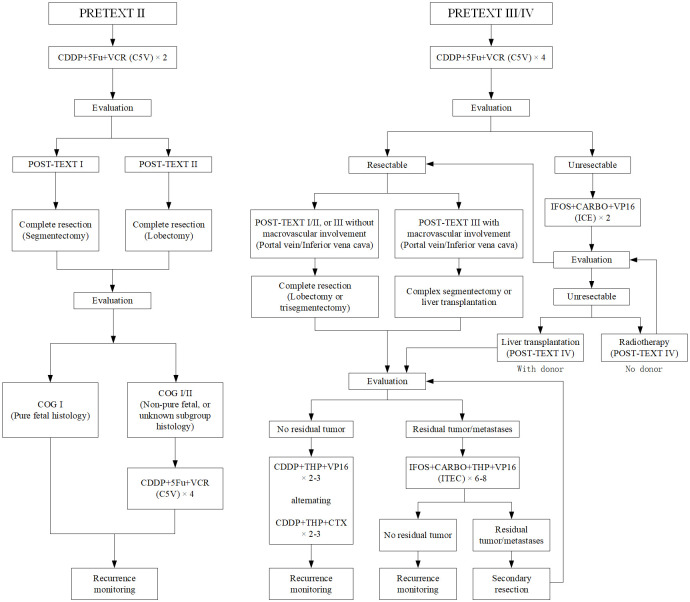
Therapeutic protocol. The comprehensive therapeutic protocol for hepatoblastoma.

All children received a combination of surgery and chemotherapy. Due to the inaccessibility of suitable donor organ, none had received liver transplantation before recurrence. Only 1 patient underwent living-donor liver transplant after tumor recurrence. Since there were no PRETEXT I children in this study, no cases had upfront resection. Children with PRETEXT II, III and IV received neoadjuvant chemotherapy before surgical resection. Two patients received local radiotherapy after failure of neoadjuvant and intensive chemotherapy. Preoperative neoadjuvant chemotherapy lasted for 2 to 4 cycles, and postoperative adjuvant chemotherapy lasted for 4 to 8 cycles. According to the treatment schema of the COG [12,19], all children were assigned to receive C5V regimen (cisplatin: 100 mg/m^2^ or 3 mg/kg on day 1; 5-fluorouracil: 600 mg/m^2^ on day 2; vincristine: 1.5 mg/m^2^ on day 2) before surgical resection. For children with PRETEXT II, 2 cycles of C5V were performed before resection. Complete segmentectomy and lobectomy were performed in children with POST-TEXT I and POST-TEXT II after neoadjuvant chemotherapy, respectively, with at least a 1cm margin. Postoperative adjuvant chemotherapy was not required if COG stage I (pure fetal histology) was assessed postoperatively, and 4 cycles of C5V regimen chemotherapy were continued if COG stage I or II (non-pure fetal or unknown subtype histology) was assessed postoperatively. For children with PRETEXT III and IV, 4 cycles of C5V were performed before resection, and the surgical plan was divided into the following two conditions: (1) If the tumor was deemed resectable after neoadjuvant chemotherapy, POST-TEXT I/II, or III children without macrovascular involvement (portal vein/Inferior vena cava) underwent lobectomy or trisegmentectomy, while POST-TEXT III children with macrovascular involvement underwent complex segmentectomy or were considered for liver transplantation. (2) If the tumor was deemed unresectable after neoadjuvant chemotherapy, 2 cycles of intensive ICE regimen (ifosfamide: 1.5 g/m^2^ on day 1 to day 5; carboplatin: 450mg/m^2^ on day 1; etoposide: 100mg/m^2^ on day 1 to day 3) were performed, and liver transplantation or radiotherapy was considered if the tumor remained unresectable at the second evaluation after intensive chemotherapy. If there was no residual tumor after surgery, postoperative chemotherapy was performed alternately with two schemas: (1) cisplatin (100 mg/m^2^ or 3 mg/kg on day 1), pirarubicin (25 mg/m^2^ on day 1 to day 3), and etoposide (100 mg/m^2^ on day 1 to day 4); (2) cisplatin (100 mg/m^2^ or 3 mg/kg on day 1), pirarubicin (25 mg/m^2^ on day 1 to day 3), and cyclophosphamide (800–1000 mg/m^2^ on day 1), with a total cycle of 4 to 6. If there were residual tumors after surgery, the chemotherapy was performed with ITEC regimen (ifosfamide: 300 mg/m^2^ on day 1 to day 2; carboplatin: 400 mg/m^2^ on day 1; pirarubicin: 25 mg/m^2^ on day 4 and day 5; etoposide: 100 mg/m^2^ on day 1 to day 5), with a total cycle of 6 to 8. Children with residual lung or brain metastatic diseases after postoperative chemotherapy were treated with secondary surgical resection (Fig 1). Granulocyte Colony-Stimulating Factor (5 μg/kg/day) was used after each cycle. Supportive care during chemotherapy includes use of amifostine (a dose of 740 mg/m^2^ was given before chemotherapy) and enteral nutritional or intravenous nutritional support. Surgical resection of the tumor was performed at the cooperative hospital. All children with recurrence underwent a second surgical resection, and the pathology was consistent with the first. In patients with CR, serum AFP was reduced to normal level prior to discontinuation of treatment, and PET/CT was performed to determine that there were no residual tumors.

### Surveillance after CR

All children with CR after treatment were monitored for tumor recurrence. In the process of surveillance, clinicians often place more emphasis on monitoring children who have high risk factors, resulting in outcome bias. To avoid this, review intervals were kept the same for all children. Within 1 year after CR, the lesions were reviewed with CT, ultrasound examination, and serum AFP every month; after 1 year of CR, serum AFP was assessed every month, whereas CT and ultrasound were performed every 3 months; after 3 years of CR, serum AFP, CT and ultrasound were assessed every 3 months. If the child developed a fever, abdominal pain, abdominal distension, or an abdominal mass during the period of CR, the serum AFP and related imaging examination would be immediately rechecked.

### Data collection and statistical analysis

All medical record data, including variable data, were obtained from the electronic medical record system of the hospital. Information that could identify individual participants was not available to the authors during or after data collection. The lost follow-up cases were retained as the truncated data and also participated in the statistics. We used means or medians to describe the continuous variables, whereas we used frequency and percentage to describe the categorical variables. We employed the Kaplan–Meier method for the univariate analysis and the log-rank test for evaluating the difference between univariate factors. We established a multivariate Cox regression model to assess the independent factors affecting recurrence and adopted the forward LR method for screening out the independent risk factors. *P* < 0.05 indicated that the difference was statistically significant. We analyzed the data with SPSS 22.0.

## Results

### General data

**Fig 2:**
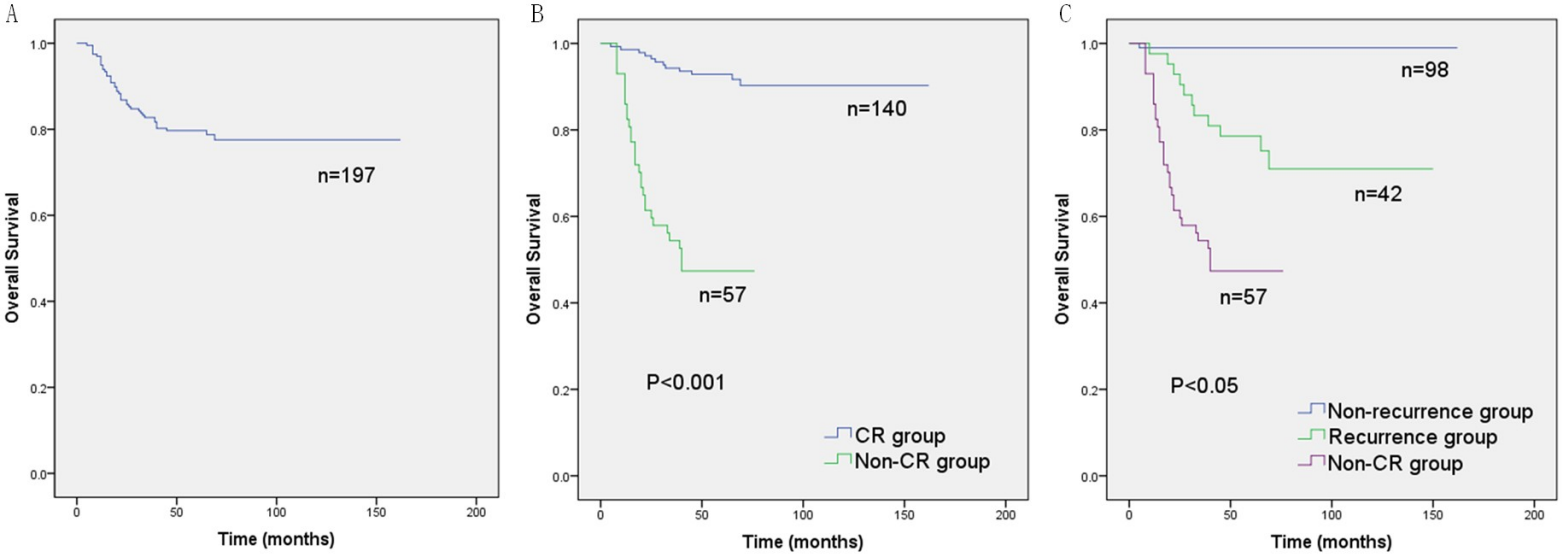
Overall survival (OS) curves of children with hepatoblastoma in each cohort. (A) OS curve of entire cohort in 197 children. (B) Comparison of OS curves between complete remission (CR) group and non-CR group. (C) Comparison of OS curves among non-recurrence group, recurrence group, and non-CR group. (Kaplan-Meier method, *P* value shown for Log-rank test between variables).

A total of 197 children with hepatoblastoma confirmed by postoperative pathology were analyzed in this study. Of these patients, 140/197 (71.1%) showed an initial CR after treatment and 57/197 (28.9%) never achieved CR during follow-up. The 5-year OS rates for the entire cohort, CR group, and non-CR group were 79.7%, 92.9%, and 47.4%, respectively. In 140 children with initial CR, the 5-year OS rates for the non-recurrence and recurrence group were 99.0% and 78.6%, respectively (Table 1 and Fig 2). Of the 57 patients in the non-CR group, 6 were PRETEXT III and 51 were PRETEXT IV. Two patients could not be resected after preoperative chemotherapy, among whom one chose radiotherapy due to lack of donor for liver transplant and the other died after abandoning further treatment. Macroscopic residues were found in 12 patients after surgical resection. The clinical course of the entire cohort was shown in S1 Fig in the supplementary materials.

**Table 1:** Comparison of OS rates in each cohort of children with hepatoblastoma.

	n	1-year OS (%)	3-year OS (%)	5-year OS (%)	*P* value
Entire cohort	197	94.9	82.7	79.7	
CR group	140	98.6	94.3	92.9	<0.001
Non-CR group	57	86.0	54.4	47.4	
Recurrence group	42	97.6	83.3	78.6	0.001
Non-CR group	57	86.0	54.4	47.4	
Non-recurrence group	98	99.0	99.0	99.0	<0.001
Non-CR group	57	86.0	54.4	47.4	
Non-recurrence group	98	99.0	99.0	99.0	<0.001
Recurrence group	42	97.6	83.3	78.6	

OS, Overall survival; CR, Complete remission.

A total of 140 children who achieved an initial CR were included in the study of recurrence factors. Of these children, 77/140 (55%) were males and 63/140 (45%) were females. The mean age at diagnosis was 2.06 ± 2.22 years, with minimum and maximum ages of 0 (at birth) and 12.58 years, respectively. Follow-up was conducted by regular outpatient visits or telephone inquiries, including symptoms, signs, tumor biomarkers, and imaging results. Follow-up was initiated when CR was achieved for the first time until recurrence occurred. The mean follow-up was 70.192 ± 2.762 months, with a median follow-up of 63.0 ± 2.241 months.

Among them, 35/140 (25.0%) had metastatic diseases at initial visit. There were 30/35 (85.7%) cases of lung metastasis, 7/35 (20.0%) cases of bone metastasis, 1/35 (2.9%) case of intracranial metastasis, 1/35 (2.9%) case of pleural metastasis, and 1/35 (2.9%) case of implantation metastases (both intestine and diaphragm involved). Multiple metastatic diseases were found in 5/35 (14.3%) cases. Lung and bone were simultaneously involved in 3/35 (8.6%) cases, lung and pleura were simultaneously involved in 1/35 (2.9%) case, while colon, diaphragm and lung were simultaneously involved in 1/35 (2.9%) case. Among the patients with lung metastasis, unifocal metastasis was found in 4/30 (13.3%) cases, while multifocal metastases were found in 26/30 (86.7%) cases. Among the patients with vascular involvement, portal vein (P+) was involved in 44/51 (86.3%) cases, vena cava or hepatic vein (V+) was involved in 6/51 (11.8%) cases, and both portal vein and hepatic vein (P+ and V+) were involved in 1/51 (2.0%) case.

The 1-year, 3-year, and 5-year RFS rates for the cohort of 140 children with CR were 77.8%, 69.8%, and 69.8%, respectively. The demographic and clinical features of the cohort are shown in Table 2.

**Table 2:** Clinical features of 140 children with initial CR.

	Total (n = 140)
RFS	
1-year RFS (%)	77.8
3-year RFS (%)	69.8
5-year RFS (%)	69.8
Recurrence [n (%)]	
Yes	42 (30.0)
No	98 (70.0)
Age (years) [n (%)]	
<3	109 (77.9)
≥3	31 (22.1)
Sex [n (%)]	
Male	77 (55.0)
Female	63 (45.0)
Pathologic type [n (%)]	
Epithelial	96 (68.6)
Mixed	44 (31.4)
PRETEXT stage [n (%)]	
II	6 (4.3)
III	57 (40.7)
IV	77 (55.0)
Metastatic disease [n (%)]	
Yes	35 (25.0)
No	105 (75.0)
Vascular involvement [n (%)]	
Yes	51 (36.4)
No	89 (63.6)
Initial resection performed [n (%)]	
Segmentectomy	2 (1.4)
Right lobectomy	67 (47.9)
Left lobectomy	25 (17.9)
Trisegmentectomy	19 (13.6)
Complex segmentectomy	27 (19.3)
Surgical margin status [n (%)]	
MacroPM	5 (3.6)
MicroPM	37 (26.4)
MicroNM	98 (70.0)
Serum AFP level (ng/mL) [n (%)]	
<1000	10 (7.1)
≥1000	130 (92.9)

CR, complete remission; RFS, recurrence-free survival; PRETEXT, PRE-Treatment EXTent of tumor; MacroPM, macroscopically positive margin; MicroPM, microscopically positive margin; MicroNM, microscopically negative margin; AFP, alpha-fetoprotein.

Of 140 children, 42 (30%) had recurrent hepatoblastoma. Among them, there were 15/42 (35.7%) cases of local recurrence, 16/42 (38.1%) cases of single organ distant recurrence, and 11/42 (26.2%) cases of combined recurrence. In the patients with local recurrence, 8/15 (53.3%) cases of right lobe, 1/15 (6.7%) case of left lobe, 1/15 (6.7%) case of caudate lobe, and 5/15 (33.3%) cases of multiple liver lobes were observed, respectively. Of the single organ distant recurrences, 15/16 (93.8%) were pulmonary and 1/16 (6.2%) was intracranial. Among the patients with combined recurrence, there were 6/11 (54.5%) cases of liver combined with lung recurrence, 3/11 (27.3%) cases of liver combined with portal vein recurrence, 1/11 (9.1%) case of liver combined with bone recurrence, and 1/11 (9.1%) case of liver, lung and portal vein combined recurrence. Of the 27 patients with distant recurrences, 12 (44.4%) initially had localized tumors at diagnosis. All recurrences occurred within 2 years from CR, and the timing of recurrence ranged from 2 to 20 months, with a mean of 8.6 ± 4.949 months and a median of 7 months. The mean level of serum AFP was 7,736.52 ± 36,300.70 ng/mL (median 586.00 ng/mL) during the time of recurrence, with the maximum and minimum values of 236,051 ng/mL and 19.4 ng/mL, respectively. Only 1/42 (2.4%) patient did not have elevated AFP at the time of tumor recurrence (AFP normal range: 0–20 ng/mL), and the histological type of the patient was epithelial (mixed fetal and embryonal subtype) after the first and second surgeries.

Of the 5 patients older than 8 years, 4 (80.0%) had epithelial histological type and 1 (20.0%) had mixed histological type. Among the 4 cases with epithelial type, there were 2 cases of pure fetal subtype, 1 case of mixed fetal and embryonal subtype, and 1 case of unknown subtype. 4/5 (80.0%) patients developed recurrences, and one patient was free of recurrence during 125 months of follow-up. The histological type of the 2 patients with AFP <100 ng/mL were epithelial, including 1 case of embryonal subtype and 1 case of unknown subtype, and no recurrence was observed during the follow-up period of 72 months and 125 months, respectively. Of the two patients with undifferentiated small cell histological type, one relapsed 5 months after CR and the other did not relapsed during the period of 99 months after initial CR. The histological type of the one patient with bone recurrence and the other with intracranial recurrence were both mixed.

### Univariate RFS analysis of risk factors for recurrence

**Fig 3:**
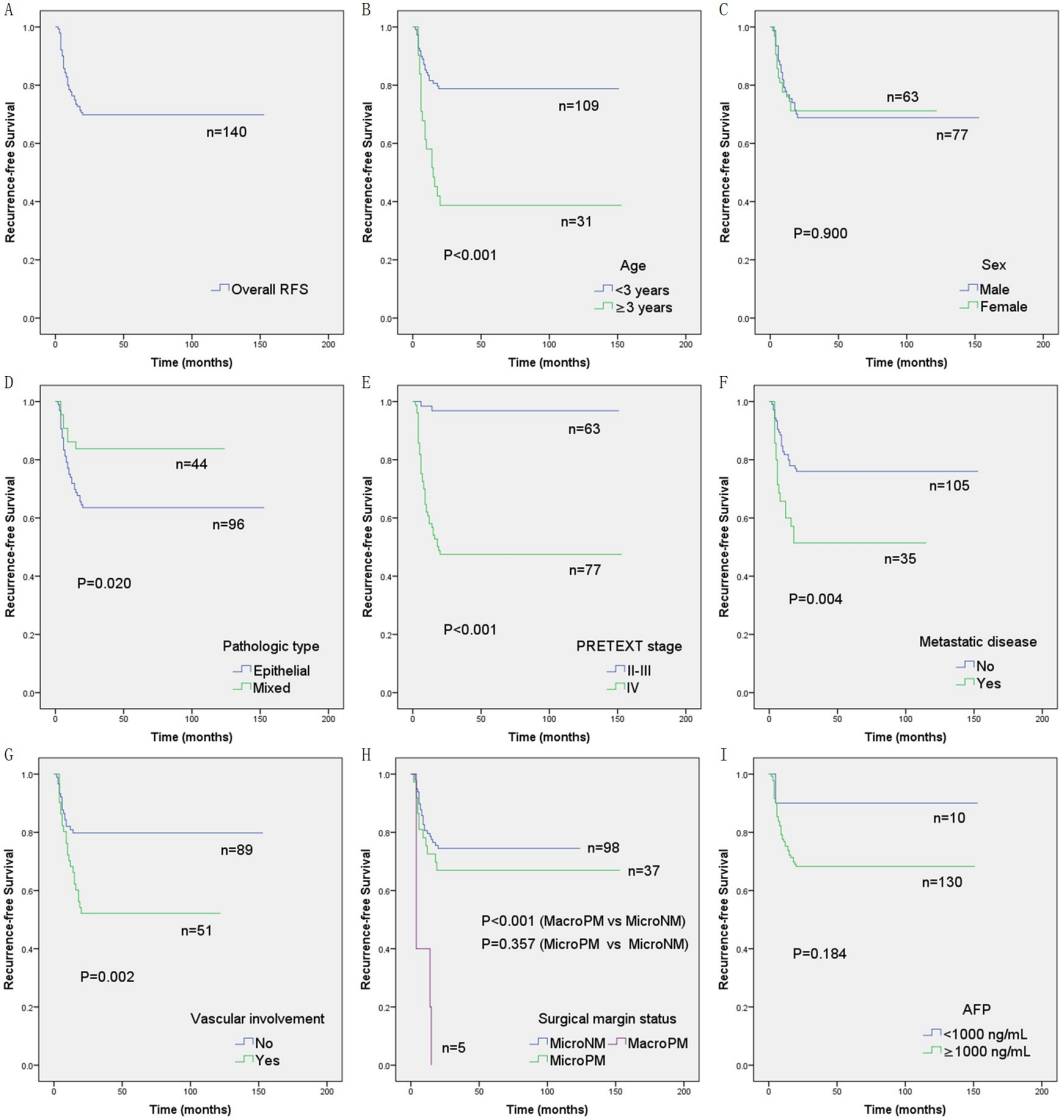
Overall recurrence-free survival (RFS) and comparisons of RFS by age, sex, pathologic type, PRETEXT stage, metastatic disease, vascular involvement, surgical margin status, and serum AFP level. (A) Overall RFS. (B) Comparison of RFS by age. (C) Comparison of RFS by sex. (D) Comparison of RFS by pathologic type. (E) Comparison of RFS by PRETEXT stage. (F) Comparison of RFS by metastatic disease. (G) Comparison of RFS by vascular involvement. (H) Comparison of RFS by surgical margin status, including macroscopically positive margin (MacroPM), microscopically positive margin (MicroPM), and microscopically negative margin (MicroNM). (I) Comparison of RFS by serum AFP level. (Kaplan-Meier method, *P* value shown for Log-rank test between variables).

We statistically analyzed the age, sex, PRETEXT stage, pathologic type, metastatic disease, serum AFP level, vascular involvement, and surgical margin status of all children with initial CR. The 1-year, 3-year, and 5-year RFS rates for each cohort are shown in Table 3, and the plotted RFS curves are shown in Fig 3. The results showed that the RFS rate of children younger than 3 years, with mixed pathological type, PRETEXT II and III, without metastatic disease, without vascular involvement, and MicroNM was significantly higher than that of children older than 3 years, with epithelial pathological type, PRETEXT IV, metastatic disease, vascular involvement, and MacroPM (*P* < 0.001, = 0.020, < 0.001, = 0.004, = 0.002, and < 0.001, respectively). There were no statistically significant differences in RFS rate for sex, serum AFP levels, and microscopic surgical margin status (*P* = 0.900, 0.184, and 0.357, respectively).

**Table 3:** Comparison of RFS in each cohort of 140 children with initial CR.

	Total (n = 140)	1-year RFS (%)	3-year RFS (%)	5-year RFS (%)	*P* value
Age (years)					
<3	109	83.4	78.8	78.8	<0.001
≥3	31	58.1	38.7	38.7	
Sex					
Male	77	76.6	68.8	68.8	0.900
Female	63	76.0	71.2	71.2	
Pathologic type					
Epithelial	96	74.0	63.5	63.5	0.020
Mixed	44	86.1	83.8	83.8	
PRETEXT stage					
II-III	63	98.4	96.8	96.8	<0.001
IV	77	60.7	47.5	47.5	
Metastatic disease					
Yes	35	65.7	51.4	51.4	0.004
No	105	81.8	76.0	76.0	
Vascular involvement					
Yes	51	68.2	52.2	52.2	0.002
No	89	80.9	79.8	79.8	
Surgical margin status					
MacroPM	5	40.0	0	0	<0.001(MacroPM vs MicroNM)
MicroPM	37	72.5	67.0	67.0	
MicroNM	98	79.6	74.5	74.5	0.357(MicroPM vs MicroNM)
Serum AFP level (ng/mL)					
<1000	10	90.0	90.0	90.0	0.184
≥1000	130	76.8	68.3	68.3	

CR, complete remission; RFS, recurrence-free survival; PRETEXT, PRE-Treatment EXTent of tumor; MacroPM, macroscopically positive margin; MicroPM, microscopically positive margin; MicroNM, microscopically negative margin; AFP, alpha-fetoprotein.

### Multivariate RFS analysis of risk factors for recurrence

We assessed age, sex, PRETEXT stage, pathologic type, metastatic disease, serum AFP level, vascular involvement, and surgical margin status of 140 children who achieved initial CR with a multivariate Cox regression model. After variable screening, the independent risk factors for recurrence after CR in the model were age ≥3 years, PRETEXT IV, and metastatic disease (*P* < 0.05), whereas sex, pathologic type, serum AFP level, vascular involvement, and surgical margin status had no significant effect on recurrence after CR (*P* > 0.05) (Table 4). The hazard ratio for age ≥3 years, PRETEXT stage IV, and metastatic disease was 2.561, 16.822, and 2.176, respectively.

**Table 4:** Cox proportional hazard model for the independent risk factors of recurrence in 140 children with initial CR.

	n	Hazard ratio	95% CI	*P* value
**Variables in the model**				
Age (years)				
<3	109	Reference group	Reference group	0.004
≥3	31	2.561	1.362–4.816	
PRETEXT stage				
II-III	63	Reference group	Reference group	<0.001
IV	77	16.822	4.012–70.543	
Metastatic disease				
Yes	35	2.176	1.150–4.117	0.017
No	105	Reference group	Reference group	
**Variables excluded from the model**				
Sex				
Male	77			0.687
Female	63			
Pathologic type				
Epithelial	96			0.057
Mixed	44			
Vascular involvement				
Yes	51			0.296
No	89			
Surgical margin status				
MacroPM	5			0.148
MicroPM	37			
MicroNM	98			
Serum AFP level (ng/mL)				
<1000	10			0.220
≥1000	130			

CR, complete remission; RFS, recurrence-free survival; MacroPM, macroscopically positive margin; MicroPM, microscopically positive margin; MicroNM, microscopically negative margin; AFP, alpha-fetoprotein.

## Discussion

In recent years, with the introduction of risk stratification methods for hepatoblastoma and improvements in treatment regimens, EFS and OS rates in children with hepatoblastoma have improved significantly. Many children achieve CR after a combination of surgery and chemotherapy. Due to the lack of understanding of the risk of recurrence, a unified screening method is often used for monitoring children after CR. Such untargeted re-examination surveillance could delay the diagnosis of recurrent hepatoblastoma in children at high risk of recurrence, while leading to excessive examinations of children at very low risk of recurrence. Moreover, some well-defined factors that can affect long-term survival, such as age, serum AFP, PRETEXT stage, and vascular involvement, are not necessarily the risk factors for recurrence after remission. Although clinicians can still use these risk factors to assess long-term survival in children, there are limitations in assessing the risk of recurrence, and the predictions are not reliable. Therefore, it is necessary to identify the factors that influence tumor recurrence to help establish a classification system for the recurrence risk of hepatoblastoma. If factors causing recurrence are clearly identified and a risk stratification system for recurrence after CR is established, the long-term prognosis management of children with CR can be more scientifically conducted.

By plotting the survival curve, it can be found that the 3-year OS rate in children with relapsed hepatoblastoma in our study (83.3%) was significantly higher than that in patients collected between 1990 and 2004 in the SIOPEL study (43%) [6]. Therefore, we can infer that, with the establishment of risk classification and the improvement of treatment regimens, there has been a significant OS improvement not only in the overall patients with hepatoblastoma (from 27% reported in the 1990s to 79% reported in 2013), but also in the patients with post-remission recurrence. However, the relatively high recurrence rate is still one of the important factors that seriously affect the quality of life in children with hepatoblastoma. By comparing the OS rates of the non-recurrence, recurrence and non-CR groups, we found that the 5-year OS rate in the non-recurrence group was significantly higher than that in the recurrence group; however, even if children had a post-remission recurrence, they showed a significantly better OS than those who never achieved a remission. For these children with non-CR hepatoblastoma, there may be unknown adverse prognostic factors that need to be further confirmed in future studies.

Based on the above results of this study, Age, PRETEXT stage, metastatic disease, vascular involvement, pathologic type, and surgical margin status might be associated with recurrent hepatoblastoma after CR; meanwhile, age ≥3 years, PRETEXT IV, and metastatic disease are independent risk factors of recurrence. Analysis showed that the recurrence of hepatoblastoma was often accompanied by distant metastases and abnormally elevated serum AFP levels. The most common site of in situ recurrence was the right lobe, followed by the multiple lobes. The lung was the most common site of distant recurrence, while intracranial and bone recurrence was rare. An interesting finding among the results of our study was that all the children with recurrent hepatoblastoma had the recurrence within 2 years after CR, and those without recurrence within the first 2 years had no recurrence throughout the follow-up period. It can be inferred that children with hepatoblastoma have a negligible chance of recurrence after 2 years of CR. However, further research is needed before any definite conclusions can be drawn.

Although a large number of studies have shown that age has an influence on the prognosis of hepatoblastoma [20–22], its influence on various PRETEXT stages has not yet been determined. The SIOPEL found that an age 3–7 years and 8 years or older were predictors of a poorer prognosis in PRETEXT I/II and PRETEXT IV children, but this effect was not observed in PRETEXT III. Therefore, it was difficult to make a universal treatment recommendation based on age that applies across all PRETEXT stages [11]. When we used the same age segmentation in evaluating the impact of age on recurrence after CR, we did not conduct stratification in terms of PRETEXT due to the limited number of cases. However, in the results of the multivariate analysis, an age older than 3 years was still an independent risk factor leading to recurrence, even after adjusting for the PRETEXT variable. Our results showed that in children with PRETEXT stage II–III or stage IV, age played an important role for recurrence after CR. With the same PRETEXT stage and metastasis status, the risk of recurrence was 2.561 times higher in children older than 3 years than in those younger than 3 years. [Deleted sentence] In future studies, a large sample size is needed to determine the effect of age > 8 years on tumor recurrence.

Except for age, PRETEXT stage itself appeared to play a crucial part in recurrence. The PRETEXT staging system was originally designed by SIOPEL for the staging and basis of liver tumor risk stratification [23,24]. Based on the SIOPEL experience, good prognostic factors included a low PRETEXT at diagnosis (PRETEXT I, II, and III) whereas a poor outcome correlated with PRETEXT IV [25,26]. When evaluating its impact on recurrence using the PRETEXT staging system, the RFS rate of children with PRETEXT IV was much lower than that of children with PRETEXT II and III. PRETEXT IV was an independent risk factor for recurrence after CR, and with the same age and metastasis status, the risk of recurrence was 16.822 times higher in children with PRETEXT IV than in those with PRETEXT II and III. Thus, the PRETEXT staging system could not only be used for assessing the general prognostic outcome, such as EFS or OS, but might also be of value in the evaluation of tumor recurrence after CR.

We also analyzed the PRETEXT annotation factors of metastatic disease and vascular involvement. Metastatic disease as a poor prognosis has become the current consensus, which correlates significantly with reduced EFS and OS [2,7,9,17,26–28]. The CHIC had singled out metastatic disease as a separate risk stratification backbone [11]. Vascular involvement has also generally been believed to be an important prognostic factor related to a poorer outcome than in those without vascular involvement [7,9,27], even though some early studies showed that vascular invasion did not influence the prognosis [29]. In our study, we found that children with metastasis and vascular involvement had a significantly lower RFS than those without metastasis and vascular involvement. However, the multivariate analysis showed that only metastatic disease was an independent risk factor for recurrence. With the same age and PRETEXT stage, the risk of recurrence was 2.176 times higher in children with metastatic disease than in those without metastatic disease. As for the reliability of the results, more large-sample studies are needed for further demonstration.

The increase in serum AFP plays an important role in the diagnosis of hepatoblastoma, and the change in serum AFP value is important for predicting the outcome of children with hepatoblastoma [7,8,27,30]. A recent study has found the diagnostic value of AFP for recurrence of hepatoblastoma, showing that AFP elevation was specific at detecting relapse after CR [5]. However, no research has yet systematically studied whether AFP levels at the time of initial diagnosis impact recurrence in children after CR. According to the results of our study, AFP ≥ 1000 ng/mL is not an independent risk factor for recurrence of hepatoblastoma (AFP < 1000 ng/mL as reference), and there was no significant difference in RFS rates. Due to the insufficient number of cases with AFP < 100 ng/mL, we were unable to assess the risk of recurrence between AFP ≥ 100 ng/mL and AFP < 100 ng/mL. Therefore, this study still lacks evidence to conclude that serum AFP level has no effect on recurrence, but only that AFP ≥ 1000 ng/mL is not a risk factor for recurrence compared with AFP < 1000 ng/mL. For the 2 patients with serum AFP < 100 ng/mL, no recurrence was observed during the entire follow-up period. Therefore, a large sample study is needed to confirm whether AFP < 100 ng/mL has an influence on post-remission recurrence.

It was not until 2014 that a basic consensus was reached on the histopathologic classification criteria for hepatoblastoma [15,31,32]; thus, the pathological classification has not been included in the PRETEXT risk stratification system. Hepatoblastoma includes epithelial and mixed epithelial and mesenchymal forms. Epithelial tumors include pure fetal, embryonal, pleomorphic, macrotrabecular, small-cell, cholangioblastic, and mixed subtypes. Due to the complexity and variety of histopathological classification, it is difficult to draw a detailed and consistent conclusion regarding the impact of each subtype on prognosis. However, both early and recent studies showed that pure fetal hepatoblastoma with minimal mitotic activity is associated with better prognoses, whereas the small-cell undifferentiated subtype has poorer survival [8,29,33,34]. Since both pure fetal and small-cell undifferentiated subtypes belong to epithelial type, it is necessary to further compare the prognosis between subtypes. In our study, however, due to the fact that the majority of epithelial histopathological results indicated mixed subtypes, and some results lacked subtype classification, detailed prognostic assessment of histological subtypes was not available. Although we found that the RFS rate of the mixed type was significantly higher than that of the epithelial type, the results of multivariate analysis did not find that pathology type was an independent factor affecting recurrence.

Currently, it is almost certain that residual tumor after surgical resection is one of the adverse factors affecting the outcome of hepatoblastoma [3,28]. However, whether surgical margin status is a prognostic factor remains controversial. A growing number of studies have shown that positive margins did not influence the outcome of hepatoblastoma [35–37]. It should be noted that the evaluation of surgical margin is based on histopathologic tissue, and that a complete gross resection often involves obliteration of an extra few millimeters of tissue around the tumor specimen by electrocautery dissection [38]. Thus, the surgical margin status may not accurately reflect the true residual tumor tissue. The results of our study suggest that children with MacroPM had a significant lower RFS rate than those with MicroNM, whereas there was no significant difference in the RFS rate between MicroPM and MicroNM, which was consistent with the results of previous studies.

With improvements in the cure rate of hepatoblastoma in recent years, clinical strategies should consider how to prevent tumor recurrence after CR. A better understanding of the recurrence factors of hepatoblastoma could help establish an independent system for assessing the risk of recurrence, and provide targeted monitoring and improved initial treatment scheme for children at high risk of recurrence, thereby reducing recurrence risk and improving long-term prognosis. Although this study identified several independent risk factors including age ≥3 years, PRETEXT IV, and metastatic disease, which were associated with recurrent hepatoblastoma after CR, more research is needed for a definitive conclusion. In the near future, this may be a promising new area of research that could improve the long-term prognosis of children with hepatoblastoma by preventing tumor recurrence.

## Conclusion

Age, PRETEXT stage, metastatic disease, vascular involvement, pathologic type, and surgical margin status might be associated with recurrent hepatoblastoma after CR; meanwhile, age ≥3 years, PRETEXT IV, and metastatic disease are independent risk factors of tumor recurrence. Further research is needed on the causes of tumor recurrence, which may improve the long-term outcomes of children with hepatoblastoma.

## Supporting information

S1 Checklist STROBE checklist. (DOCX)

S1 Fig Clinical course of 197 children with hepatoblastoma. (PDF)

S2 Fig Protocol. (PDF)

S1 Table General information of 189 patients not initially treated in the hospital. (DOCX)
