# Supplementary material for: Factors influencing recurrence after complete remission in children with hepatoblastoma: A 14-year retrospective study in China
Source: PLoS One. 2021 Nov 29;16(11):e0259503. doi: 10.1371/journal.pone.0259503 (PMC8629180; doi:10.1371/journal.pone.0259503)
Supplement: S1 Fig — (PDF) [file pone.0259503.s002.pdf]

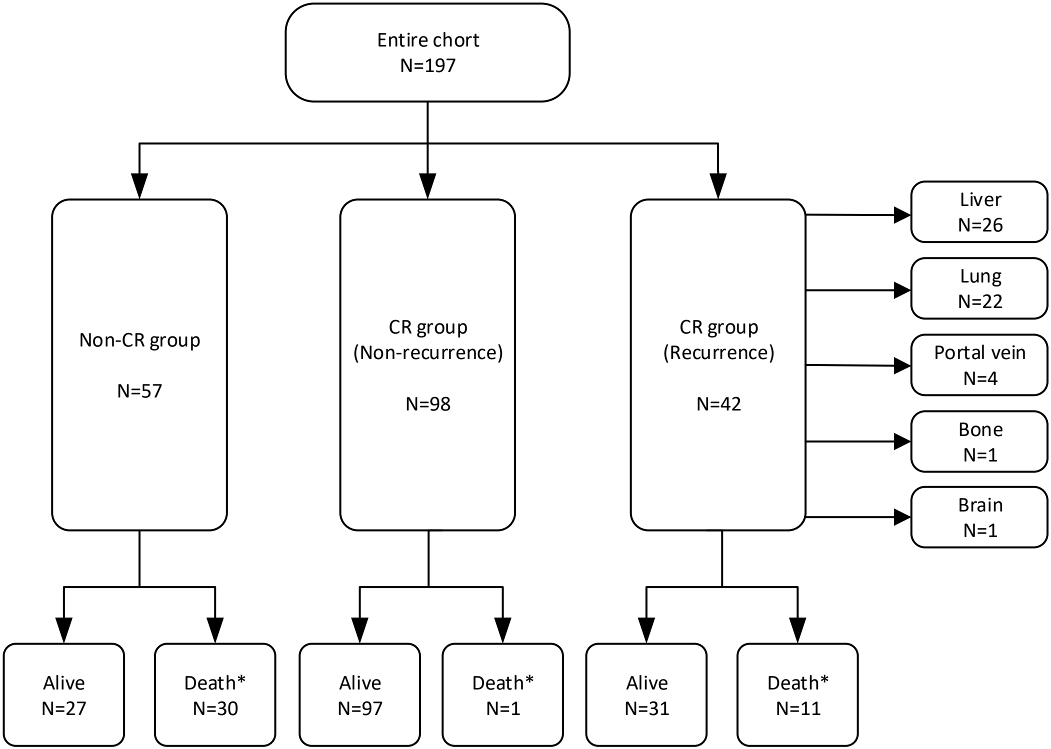

S1 Fig. Clinical course of 197 children with hepatoblastoma.
*One case in non-recurrence group died of accident. Eleven cases in recurrence group and 30 cases in non-CR group died of disease.
