# Supplementary figures and images for: Factors influencing recurrence after complete remission in children with hepatoblastoma: A 14-year retrospective study in China
Source: PLoS One. 2021 Nov 29;16(11):e0259503. doi: 10.1371/journal.pone.0259503 (PMC8629180; doi:10.1371/journal.pone.0259503)

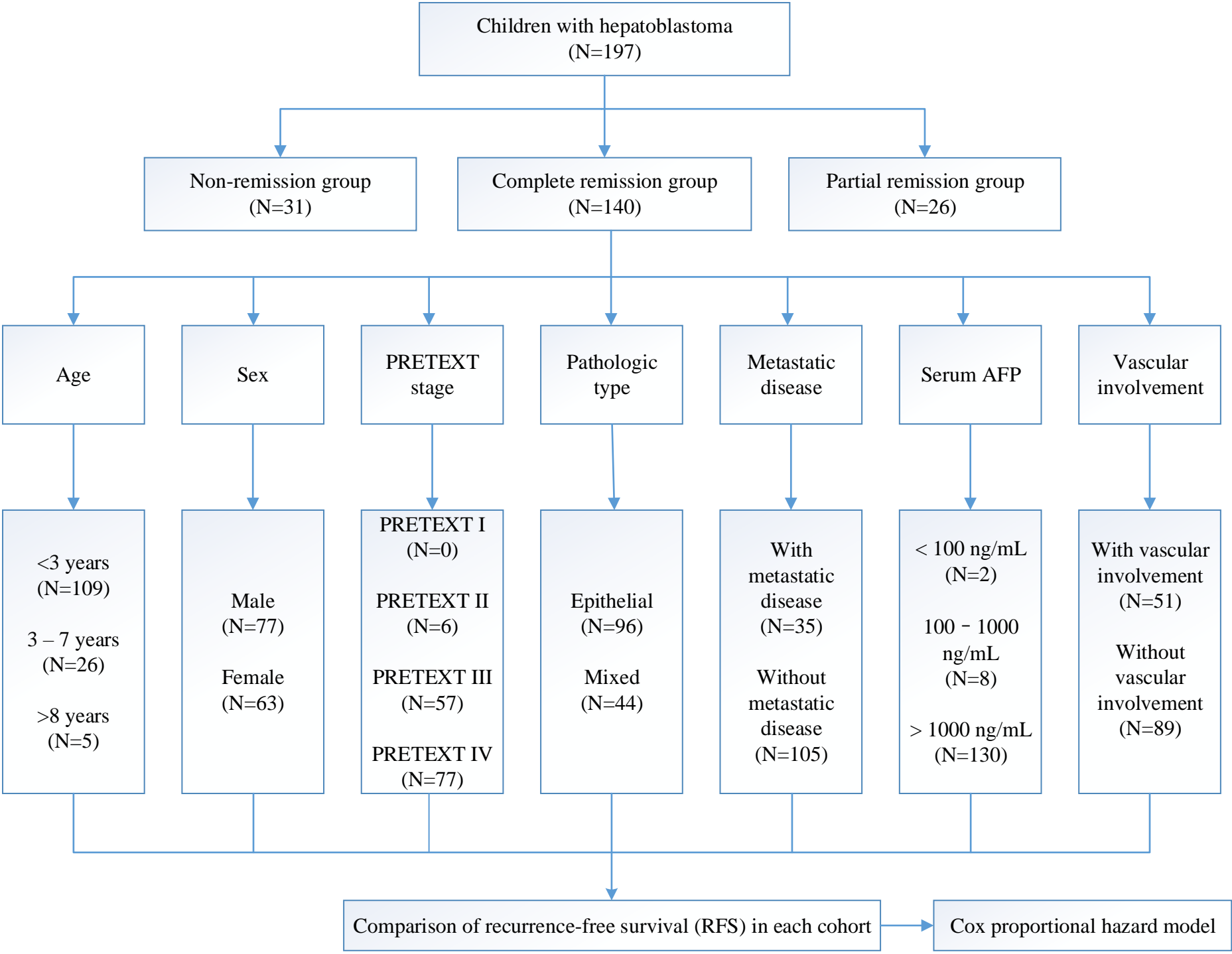

S2 Fig. Protocol

Supplement: S2 Fig — (PDF) [file pone.0259503.s003.pdf]
