# Supplementary material for: Factors influencing recurrence after complete remission in children with hepatoblastoma: A 14-year retrospective study in China
Source: PLoS One. 2021 Nov 29;16(11):e0259503. doi: 10.1371/journal.pone.0259503 (PMC8629180; doi:10.1371/journal.pone.0259503)
Supplement: S1 Table — (DOCX) [file pone.0259503.s004.docx]

**S1 Table. General information of 189 patients not initially treated in the hospital**

|  | n (%) |
| --- | --- |
| State of illness at admission |  |
| Recurrence | 115 (60.8) |
| Progression | 74 (39.2) |
|  |  |
| Clinical outcome |  |
| Alive | 122 (64.6) |
| Death | 67 (35.4) |
|  |  |
| Unknown information |  |
| Remission time | 81 (70.4) |
| Recurrence time | 16 (13.9) |
| PET/CT | 103 (54.5) |
| Initial serum AFP | 64 (33.9) |
| PRETEXT stage | 62 (32.8) |
| Chemotherapy regimen | 29 (15.3) |
| Chemotherapy cycle | 11 (5.8) |
| Resection performed | 59 (31.2) |
| Histological type | 33 (17.5) |
|  |  |
| Informal treatment |  |
| Upfront resection without chemotherapy (PRETEXT III/IV) | 47 (24.9) |
| Irregular chemotherapy regimen or cycle | 81 (42.9) |
| Unproven anti-tumor herbal remedy | 42 (22.2) |
